# A network medicine-based approach to explore the relationship between depression and inflammation

**DOI:** 10.3389/fpsyt.2023.1184188

**Published:** 2023-07-10

**Authors:** Xiaonan Hu, Huaxin Pang, Jia Liu, Yu Wang, Yifang Lou, Yufeng Zhao

**Affiliations:** ^1^Data Center of Traditional Chinese Medicine, Chinese Academy of Traditional Chinese Medicine, Beijing, China; ^2^School of Computer and Information Technology, Beijing Jiaotong University, Beijing, China; ^3^Institute of Acupuncture and Moxibustion, Chinese Academy of Traditional Chinese Medicine, Beijing, China

**Keywords:** depression, inflammation, network medicine, medical treatments, gene

## Abstract

**Background:**

Depression is widespread global problem that not only severely impacts individuals’ physical and mental health but also imposes a heavy disease burden on nations and societies. The role of inflammation in the pathogenesis and pathophysiology of depression has received much attention, but the precise relationship between the two remains unclear. This study aims to investigate the correlation between depression and inflammation using a network medicine approach.

**Methods:**

We utilized a degree-preserving approach to identify the large connected component (LCC) of all depression-related proteins in the human interactome. The LCC was deemed as the disease module for depression. To measure the association between depression and other diseases, we calculated the overlap between these disease protein modules using the Sab algorithm. A smaller Sab value indicates a stronger association between diseases. Building on the results of this analysis, we further explored the correlation between inflammation and depression by conducting enrichment and pathway analyses of critical targets. Finally, we used a network proximity approach to calculate drug-disease proximity to predict the efficacy of drugs for the treatment of depression. We calculated and ranked the distances between depression disease modules and 6,100 drugs. The top-ranked drugs were selected to explore their potential for treating depression based on the hypothesis that their antidepressant effects are related to reducing inflammation.

**Results:**

In the human interactome, all depression-related proteins are clustered into a large connected component (LCC) consisting of 202 proteins and multiple small subgraphs. This indicates that depression-related proteins tend to form clusters within the same network. We used the 202 LCC proteins as the key disease module for depression. Next, we investigated the potential relationships between depression and 299 other diseases. Our analysis identified over 18 diseases that exhibited significant overlap with the depression module. Where S_AB_ = −0.075 for the vascular disease and depressive disorders module, S_AB_ = −0.070 for the gastrointestinal disease and depressive disorders module, and S_AB_ = −0.062 for the endocrine system disease and depressive disorders module. The distance between them S_AB_ < 0 implies that the pathogenesis of depression is likely to be related to the pathogenesis of its co-morbidities of depression and that potential therapeutic approaches may be derived from the disease treatment libraries of these co-morbidities. Further, considering that the inflammation is ubiquitous in some disease, we calculate the overlap between the collected inflammation module (236 proteins) and the depression module (202 proteins), finding that they are closely related (S_di_ = −0.358) in the human protein interaction network. After enrichment and pathway analysis of key genes, we identified the HIF-1 signaling pathway, PI3K-Akt signaling pathway, Th17 cell differentiation, hepatitis B, and inflammatory bowel disease as key to the inflammatory response in depression. Finally, we calculated the *Z*-score to determine the proximity of 6,100 drugs to the depression disease module. Among the top three drugs identified by drug-disease proximity analysis were Perphenazine, Clomipramine, and Amitriptyline, all of which had a greater number of targets in the network associated with the depression disease module. Notably, these drugs have been shown to exert both anti-inflammatory and antidepressant effects, suggesting that they may modulate depression through an anti-inflammatory mechanism. These findings demonstrate a correlation between depression and inflammation at the network medicine level, which has important implications for future elucidation of the etiology of depression and improved treatment outcomes.

**Conclusion:**

Neuroimmune signaling pathways play an important role in the pathogenesis of depression, and many classes of antidepressants exhibiting anti-inflammatory properties. The pathogenesis of depression is closely related to inflammation.

## 1. Introduction

Depression is a severe mental system disorder that afflicts approximately 300 million people worldwide, and depression is characterized by high prevalence, high disability, and high suicide rates (1). Depression imposes a heavy burden of illness on individuals and society, and it has become the leading cause of mental health-related disease burden worldwide (2). There are few clinically available medications for depression, and the low cure rate, poor compliance, and high relapse rate have caused great distress to patients, their families, and society (3). Antidepressant drugs are commonly employed in clinical settings as a primary means of treating depression. However, despite this approach, only about one-third of patients ultimately exhibit positive responses to these interventions. Furthermore, while such drugs do provide some measure of therapeutic efficacy, their high cost and significant risk for side effects present obstacles to broader application (4). Therefore, there is an urgent need to discover new and better treatments. However, the etiology of depression remains a mystery, which is a severe obstacle to providing better clinical treatment options for depressed patients. Considerable human and material resources have been devoted to research on the etiology and treatment of depression. The role of neuroimmune in the pathogenesis and pathophysiology of depression has received much attention. Studies suggest that immune activation and cytokine production may be associated with depression and that cytokines are signaling proteins that mediate and regulate immune responses and inflammation. Many clinical studies have reported a correlation between depressive symptoms and concentrations of pro-inflammatory factor cells. Moreover, it has been shown that pharmacological anti-inflammatory interventions can successfully reduce both depressive symptoms and pro-inflammatory cytokine concentrations. This suggests a bidirectional causal relationship likely exists between inflammation and the onset of depressive symptoms (5). Whether inflammatory markers can serve as biomarkers of depression still needs to be investigated in depth. Here, we utilized the ideas and methods of human proteome-based network medicine analysis in (6) in the disease genetics and pharmacogenomics to explore the relationship between depression and inflammation. Compared with the previous researches about the depression and the inflammation, more relationships were introduced into our research, including disease-disease relation, disease-inflammation relation, drug-disease relation, drug-anti-inflammation relation and so on. Not only interpret the neuroimmunological mechanism of depression pathogenesis, but also provide hypotheses and ideas for treating and rationalizing depression. Overall, our studies have contributed to advancing the in-depth understanding of the relationship between neuroinflammation and depression. By utilizing techniques from networked medicine, we have simplified the complexity of traditional laboratories, improved research efficiency, and reduced research costs. These advantages have enabled researchers in the field of depression and inflammation to discover new treatments and innovative strategies more quickly. We trust that our study offers promising solutions for further development in this field.

## 2. Methods

### 2.1. Target screening for depression

To get the targets relevant to depression, we searched the MeSH^1^ database for medical keywords related to depression. Then we used the medical keyword in GeneCards^2^ and CTD^3^ databases Depression-related risk genes were retrieved. Because the Score value in the Genecards and CTD databases is an important indicator reflecting the correlation between the target and the disease, the target more closely related to depression is found based on the Score greater than the median as the screening criterion. Then 239 depression targets were screened to be standardized with the criterion and rule of the Uniprot^4^ database after duplicates were deleted. Finally, we localized these risk genes to the human protein interaction network and constructed a disease module for depression. Since different factors or pathways often contribute to the development of diseases, we utilized enrichment analysis to categorize the differential genes or substances based on their functions, in order to establish functional and phenotypic correlations.

### 2.2. Disease co-morbidity measurement

The comorbidity of diseases often indicates the existence of shared molecular mechanisms in their etiology, and studying disease co-occurrence is crucial for accurate diagnosis, effective treatment, and better prognosis (7). Therefore, before investigating the relationship between depression and inflammation, we first explored diseases that overlap with depression modules in the human protein interaction network (HPI network). The used HPI network comes from the literature (6). Our aim was to examine the connection between depression comorbidities and inflammation by taking into account existing clinical studies. Here we use a corpus of all 299 diseases defined by the Medical Subject Headings (MeSH) ontology, collected and compiled by the author Barabási (8) to explore the overlapping relationship between these 299 disease proteins and the depression disease module. We utilized the S_AB_ (A is a sub-network of the depression disease proteins, B is one of other 299 diseases proteins) metric to evaluate the network-based overlap between the protein set of different diseases, where S_AB_ < 0 signals a network-based overlap between the depression disease A and the gene associated with disease B. Next, S_AB_ was defined as follows:


$$S_{\mathrm{AB}}=\langle d_{\mathrm{AB}}\rangle -\frac{\langle d_{\mathrm{AA}}\rangle +\langle d_{\mathrm{BB}}\rangle }{2}$$

Where $\langle d_{\mathrm{AB}}\rangle$ presents the average of the shortest path distance between nodes in the depression targets A and nodes in the other disease targets B. The equation of $\langle d_{\mathrm{AB}}\rangle$ is:


$$\langle d_{\mathrm{AB}}\rangle =\frac{1}{\left||B|\right|}\underset{j\in B}{\sum }\min_{i\in A}d\left(i,j\right),$$

where d(i,j) is the length value of the shortest path between protein *i* of depression disease and protein *j* of B disease in the human protein interaction network (HPI network). ||B|| presents the number of proteins in B disease. Similarly, the definition of $\langle d_{\mathrm{AA}}\rangle$ is:


$$\langle d_{\mathrm{AA}}\rangle =\frac{1}{\left||A|\right|}\underset{j\in A}{\sum }\min_{i\in A}d\left(i,j\right).$$

### 2.3. Network-based enrichment and pathway analysis

We performed enrichment and pathway analysis of the screened vital targets based on a network medicine approach to decipher the inflammatory mechanisms of depression. We constructed the depression disease module based on the human protein interaction network and used the algorithm of network proximity (8) to explore the relationship between the disease module and inflammation critical targets in the network. The screened essential genes were then enriched to analyze the possible pathway of the neuroimmune mechanism of depression occurrence based on the important relevant disease ontology and pathways in the enrichment results. The functional annotation of the screened key targets could be obtained based on the String database.^5^ The screened key genes were further used to analyze the biological processes, molecular functions, cellular components, and signaling pathways. We expect to discover the new role of inflammatory pathways in the pathogenesis of depression. Essential cytokines may be identified to serve as biological markers for the diagnosis of depression. It will be helpful to provide new reference ideas and a basis for future clinical diagnosis of depression, and effectively predict the effect of antidepressant drug treatment. The better treatment plan promptly can be selected for the depressed patient based on his individualized characteristics.

### 2.4. Drug-disease proximity

Network-based can be used as a valid tool to explore the link between different diseases and existed drugs and measure the effect of drug treatment (9). Therefore, similar to the measurement in (6, 9), drug-disease proximity of the top-ranked drugs were used to analyze their action mechanisms, which could reveal the anti-inflammatory effects of antidepressants in the clinic as well as the potential antidepressants from the existed drugs. Since the DrugBank database provided the drug-target information and drug indication information (10), we employ the network proximity approach (6, 9) to conduct the quantitative analysis between 6,100 drugs targets collected by (6) and the depression disease module. In this method, the *Z*-value metric is used to evaluate the proximity between a disease and a drug. The process of obtaining the *Z*-value first calculates the mean value of all the closet path lengths between drug targets and diseases to get the distance *d_c_*. Specifically, given the set of depression proteins set *A* and the set of drug targets *T*, we calculate the distance $d_{c}\left(A,T\right)$ by integrating the shortest path length between nodes s and t in the network, as follows:


$$d_{c}\left(A,T\right)=\frac{1}{\left||T|\right|}\underset{t\in T}{\sum }\min_{a\in A}d\left(a,t\right)$$

However, the set sizes of various drugs are different, which leads to bias and unfair comparison, if we only use *d_c_*. as the proximity without other processes. Therefore, we created a reference distance distribution corresponding to the expected distances between two randomly selected groups of proteins matching the size and the degrees of the original depression proteins and drug targets in the network. The reference distance distribution was generated by calculating the distance between these randomly selected groups, a procedure repeated 100 times. The mean $\mu_{d_{c}\left(A,T\right)}$ and S.D. $\delta_{d_{c}\left(A,T\right)}$ of the reference distribution were used to convert an observed distance to a normalized distance, defining the proximity measure:


$$Z\left(A,T\right)=\frac{d_{c}\left(A,T\right)-\mu_{d_{c}\left(A,T\right)}}{\delta_{d_{c}\left(A,T\right)}}$$

Obviously, the smaller the *Z*-value, the better the efficacy of the drug and the higher the ranking of the drug. To assess their expected efficacy against depression, we performed a ranking of these drugs and conducted the common analysis. We found that most of the FDA-approved antidepressants were ranked highly, presenting that calculating the drug-disease proximity (*Z*-value) has practical significance. More interestingly, we found that these top-ranked drugs often have anti-inflammatory effects in clinical treatment of corresponding diseases. This implies that anti-inflammatory strategies can alleviate depressive symptoms to some extent. In addition, some drugs with similar disease modules as depression rank high on the list. We speculate that they may have a positive effect on the treatment of depression, although more clinical trials are needed to determine the results.

## 3. Results

The clinical diagnosis of depression is not only based on the patient’s clinical manifestations but also includes scale evaluation and relevant physical and chemical inspection (11). However, for some patients with atypical symptoms of depression, specific diagnostic indicators may be lacking (12). Therefore, it is important to accurately identify the pathogenesis of depression and to clarify the etiology of depression for both differential diagnosis and therapeutic care of depressed patients. A large body of clinical evidence suggests that neuroimmune plays an important role in the pathogenesis of depression and that the interaction of the central nervous system and inflammatory pathways are relevant to its development (13, 14). In the medical field, the concept of inflammation is frequently used but its clinical meaning can be ambiguous and dependent on the specific clinical context (15). One commonly employed strategy in drug development is “repurposing”—the process by which a known drug or treatment is applied to a new disease manifestation of interest. This approach enables rapid localization of potential therapeutic targets, substantially reducing clinical exploration time.

**Figure 1:**
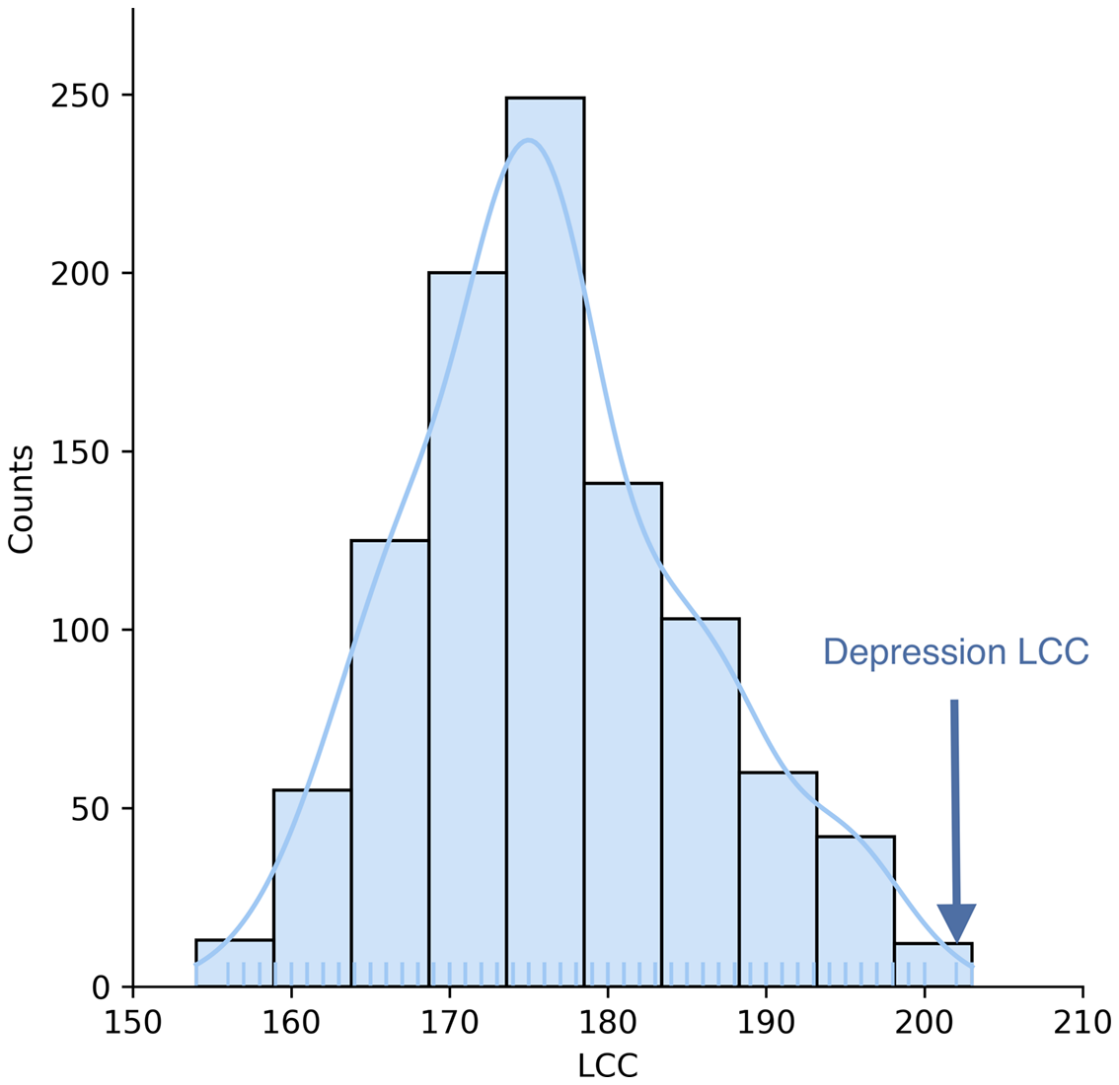
Depression disease module. Depression-targeted proteins are not randomly distributed in the human interactome but form a large connectivity component (LCC) consisting of 202 proteins and multiple small subgraphs.

**Figure 2:**
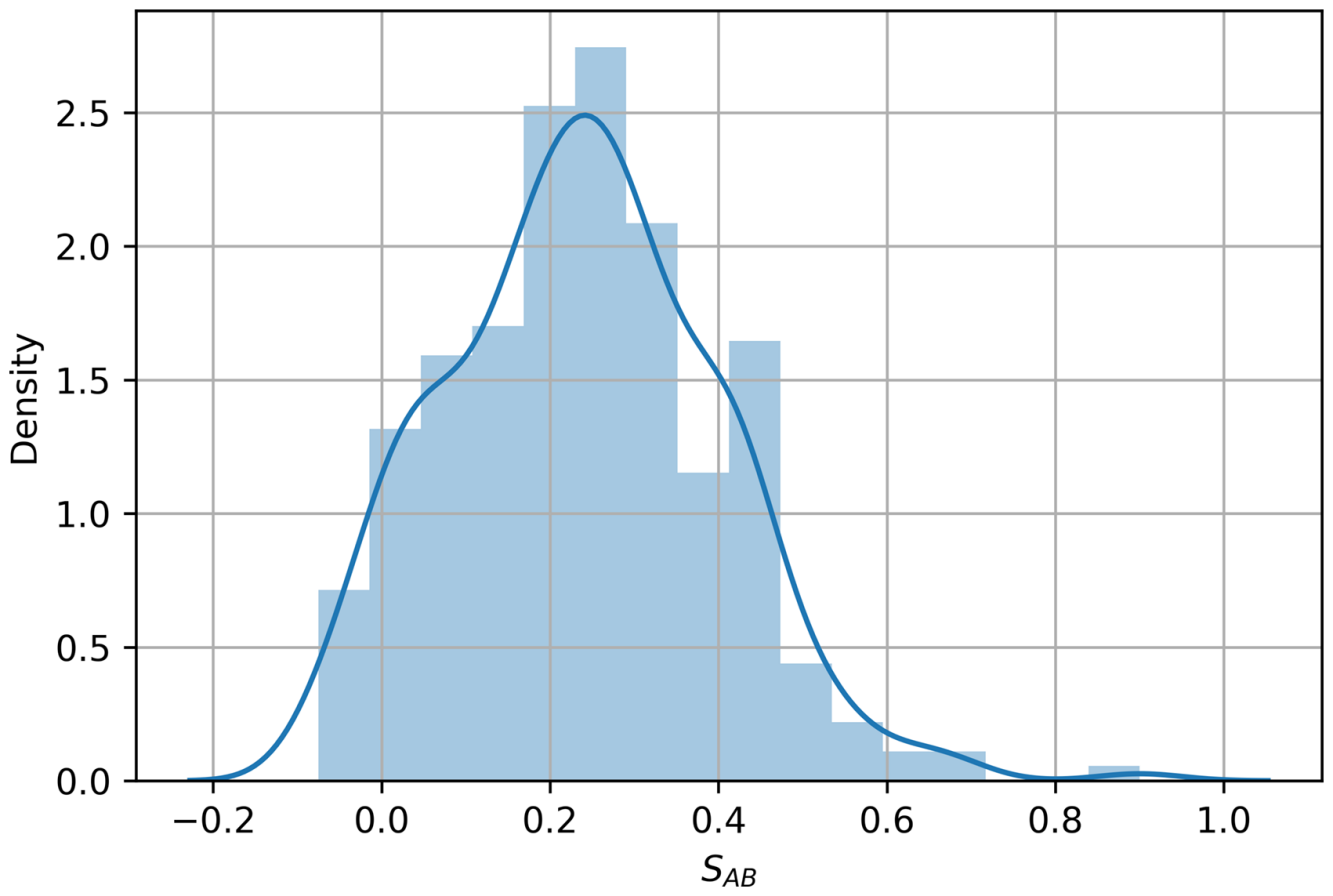
Distribution of the network overlap measure S_AB_ between 299 diseases and depression targets. S_AB_ values represent the network-based overlap between depression targets A and the genes associated with each disease B.

To search for diseases that overlap with depression in the network, we first mapped the depression disease module (consisting of 239 host protein targets) onto a human protein interaction network comprising 18,508 proteins and 332,749 physical interactions (Figure 1). The nodes in the network represent genes or their corresponding gene products, the edges refer to the connection relationship between nodes and nodes, and the network constituted by continuous phases between nodes in the network is the maximum connected module. We analyzed the distribution of overlapping relationships between the depressive disorder module and 299 other disorders, as shown in Figure 2. We found that among the relationships between disease modules and 299 disease-related proteins, depression and cardiovascular diseases, gastrointestinal diseases, and endocrine diseases have significant overlapping relationships, such as coronary heart disease, inflammatory bowel disease, and diabetes mellitus, which often appear together with depression as comorbidities (Table 1). These findings have been verified in numerous clinical studies demonstrating that patients with cardiovascular disease are more likely to experience depression than the general population, and that depressed patients have a higher risk of developing cardiovascular disease and mortality rate (16). Inflammation plays a crucial role in the development and progression of cardiovascular disease, with inflammation acting as a trigger for the early stages of the atherosclerotic process. Patients with increased inflammatory cytokines are at an elevated risk of developing cardiovascular diseases (CVD). Additionally, numerous studies indicate that depression often co-occurs with gastrointestinal diseases such as functional dyspepsia (FD), inflammatory bowel disease (IBD), gastritis, gastric ulcer, and acute enteritis, with inflammatory cytokines exerting an important pathogenic role in the development of these disorders (17). Similarly, a growing number of studies have demonstrated the correlation between depression and metabolic diseases. Depression often co-morbid with multiple metabolic diseases, including obesity and diabetes, and the underlying mechanisms of both pathogeneses involve chronic inflammation, which strongly correlates with disease severity. In summary, by exploring the overlap of depression modules with other disease proteins, this approach reveals the comorbid features of depression to some extent. By comparing these common comorbidities, we can speculate that they share similar pathogenesis. Thus, inflammation is likely to be their shared pathogenesis. This also implies that potential treatments for depression are likely to be derived from disease-specific treatments.

**Table 1:** The top 10 where depression targets overlap with 299 other disease networks.

Number	Disease	S_AB_
1	Vascular diseases	−0.075
2	Gastrointestinal diseases	−0.070
3	Cardiovascular diseases	−0.067
4	Digestive system diseases	−0.063
5	Endocrine system diseases	−0.062
6	Neurologic manifestations	−0.050
7	Intestinal diseases	−0.045
8	Neoplasms by site	−0.028
9	Bone diseases	−0.027
10	Heart diseases	−0.024

Distance ranking of diseases that overlap with the depression disease module, where the smaller the S_AB_, the larger the overlap between the two and the higher the ranking.

### 3.1. Analysis of key genes and pathways

**Figure 3:**
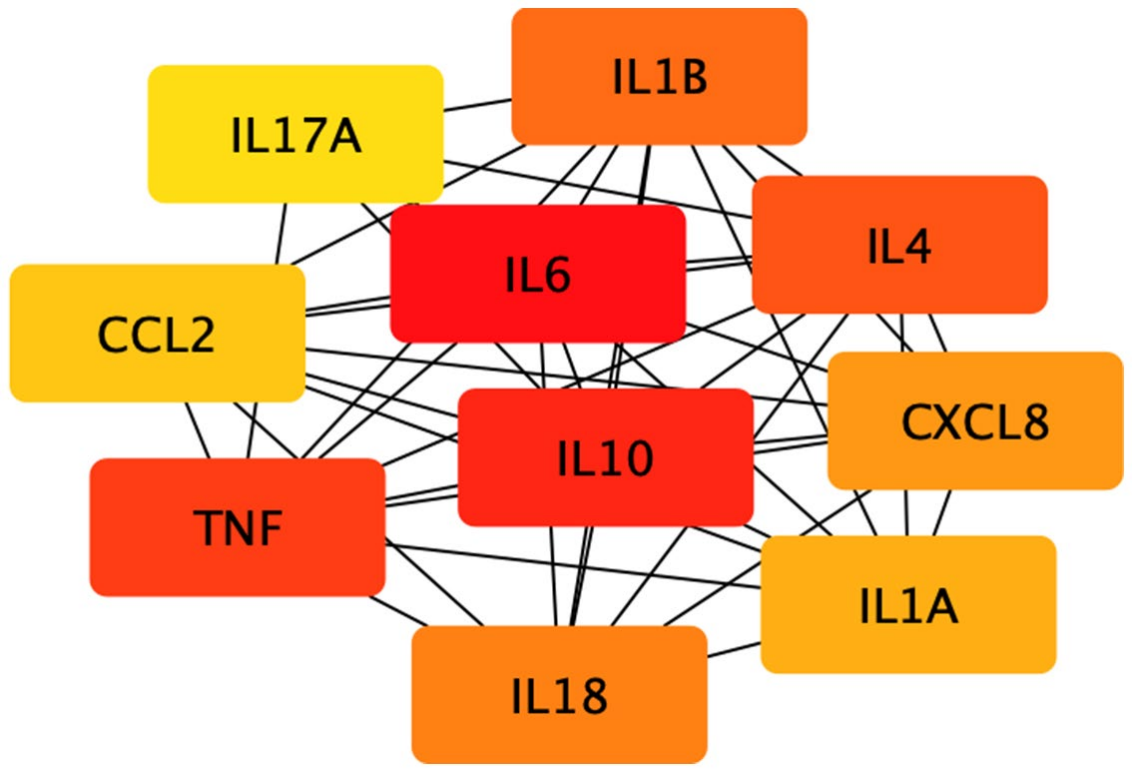
The network relationship of the first 10 key targets. The 10 key targets in the figure were screened by the MCC method.

On top of the established disease modules, using this network overlap approach, we further explored the relationship between depression and inflammation. We screened immune-related genes and their products in peripheral blood and calculated the network relationship between them and the disease module. We found that the distance between them in the network S_d_ = −0.358, i.e., the network formed by inflammation-related factors was included in the depression disease module, and the protein interaction network with 90 common targets was obtained by STRING database, with a total of 347 edges and a mean degree value of 7.71. The cytohubba tool in Cytoscape analyzed the PPI network, and the MCC method screened the top 10 key targets (Table 2). Their network relationships are shown in Figure 3. Among them, the pro-inflammatory factors TNF-α and IL-6 and the anti-inflammatory factors IL-4 and IL-10 are more central. And a large number of studies have shown that depressed patients with depression have C-reactive protein (CRP), prostaglandins, and other arachidonic acid derivatives, as well as IL-6, IL-1β and TNF-α were significantly increased (18). In addition, some data suggest that serum cytokine concentrations, including IL-6, are associated with the severity and duration of depressive illness and the effectiveness of antidepressant medication (19).

**Table 2:** The top 10 key targets were screened by the MCC method.

Rank	Name	Score
1	IL6	43,964
2	IL10	43,712
3	TNF	42,358
4	IL4	41,906
5	IL1B	41,330
6	IL18	40,442
7	CXCL8	40,440
8	IL1A	40,350
9	CCL2	40,344
10	IL17A	3,120

The PPI network was analyzed by cytohubba tool in cytoscape, and the top 10 key targets were filtered by MCC method to obtain the essential proteins in the network. Proteins with a high score tend to be critical proteins.

**Figure 4:**
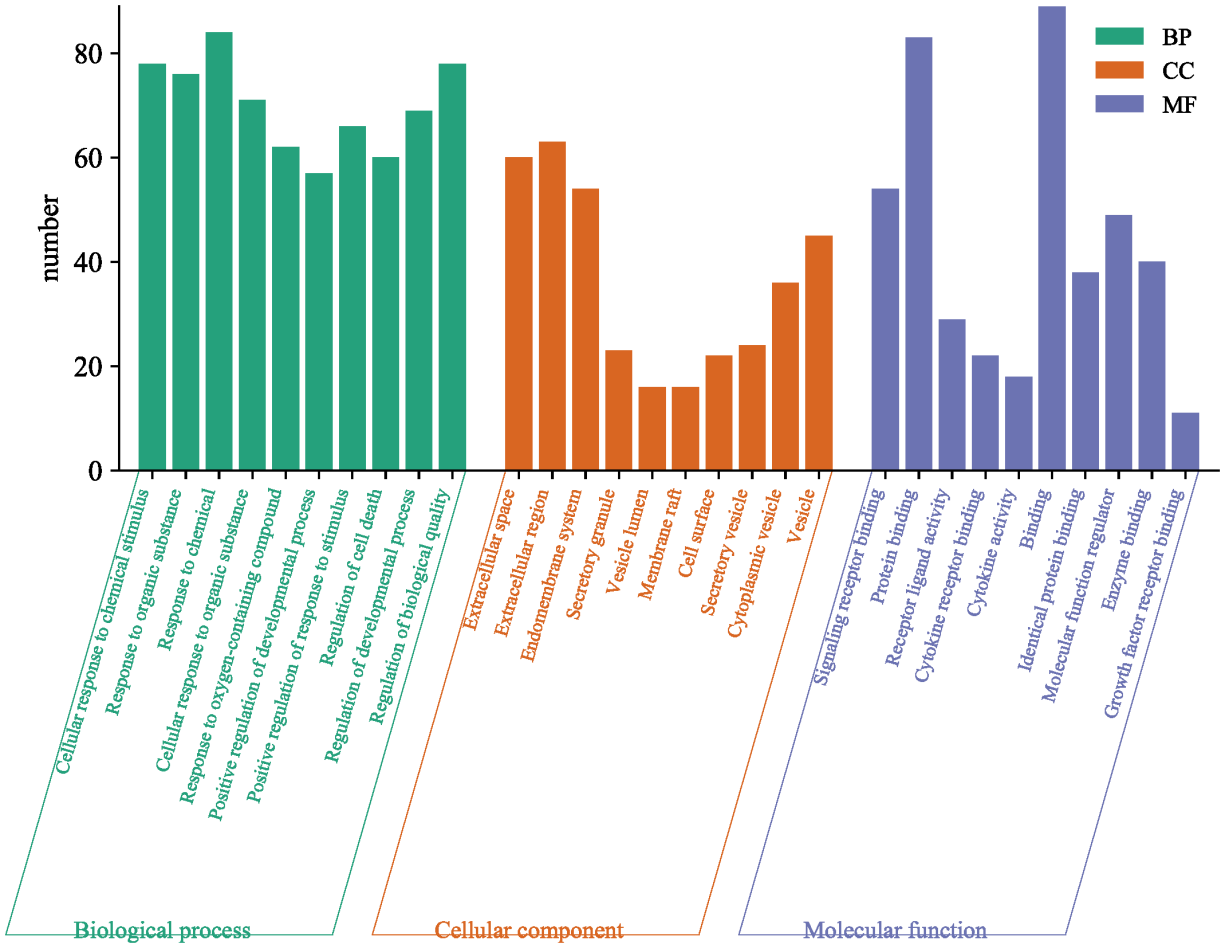
Enrichment analysis.

**Figure 5:**
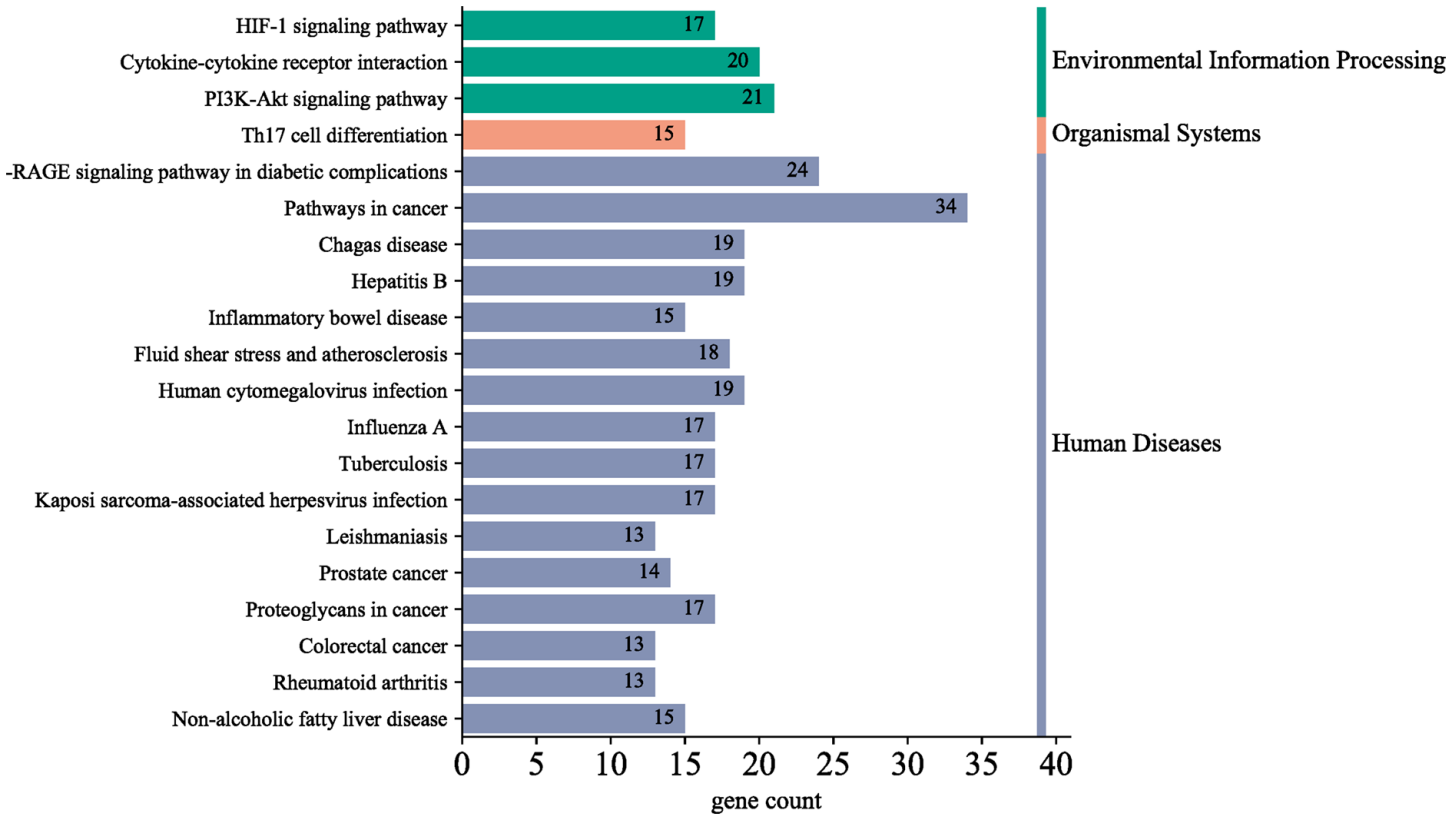
Pathway analysis.

Subsequently, we screened key genes common to both using the STRING database for functional annotation analysis (Figure 4). We found that the targets of action between depression and inflammation exert molecular functions such as regulation of cytokine activity mainly through cellular responses to chemical stimuli, organic substances, etc., based on cellular components such as extracellular gaps. The top 20 signaling pathways obtained from KEGG signaling pathway enrichment analysis are shown in Figure 5. In the pathogenesis of depression, the HIF-1 signaling pathway, PI3K-Akt signaling pathway, Th17 cell differentiation, hepatitis B, and inflammatory bowel disease are crucial in the inflammatory response. The HIF-1 signaling pathway indicates that there is an interdependent relationship between immune response and hypoxic response. Tissue hypoxia is a prominent feature of the inflammatory response. HIF is rapidly stabilized under hypoxia and is responsible for the activation of adaptive transcriptional responses, including the upregulation of metabolic factors such as vascular growth factor. In addition, studies have shown that huperzine activates the HIF-1α-VEGF signaling pathway *in vivo*, enhancing neuronal synaptic plasticity and thus exerting antidepressant effects (20, 21). In a mouse model of depression exposed to lipopolysaccharide (LPS), pioglitazone with potential anti-inflammatory effects improved depressive behavior via upregulation of the PI3K/AKT pathway (22). In clinical practice, various drugs, such as saffron and NGR1, have been shown to improve depressive behavior through the PI3K/AKT pathway (23, 24). Th17 cell differentiation may, to some extent, reflect the risk of depression in patients. Although it has been clinically shown that the signature cytokine interleukin 17A (IL-17A) produced by Th17 cells does not significantly correlate with the severity of depression, Th17 cells are involved in the gut-brain axis to mediate the stress response, possibly by promoting neuroinflammation, microglia, and astrocyte activation thereby neuronal damage triggering depressive symptoms. Therefore, Th17 cells may be a promising target for the treatment of depression (25–27). In conclusion, multiple signaling pathways are intertwined in the pathogenesis of depression, with the neuroimmune system signaling pathway playing an important role.

### 3.2. Anti-inflammatory effect analysis of antidepressants

**Figure 6:**
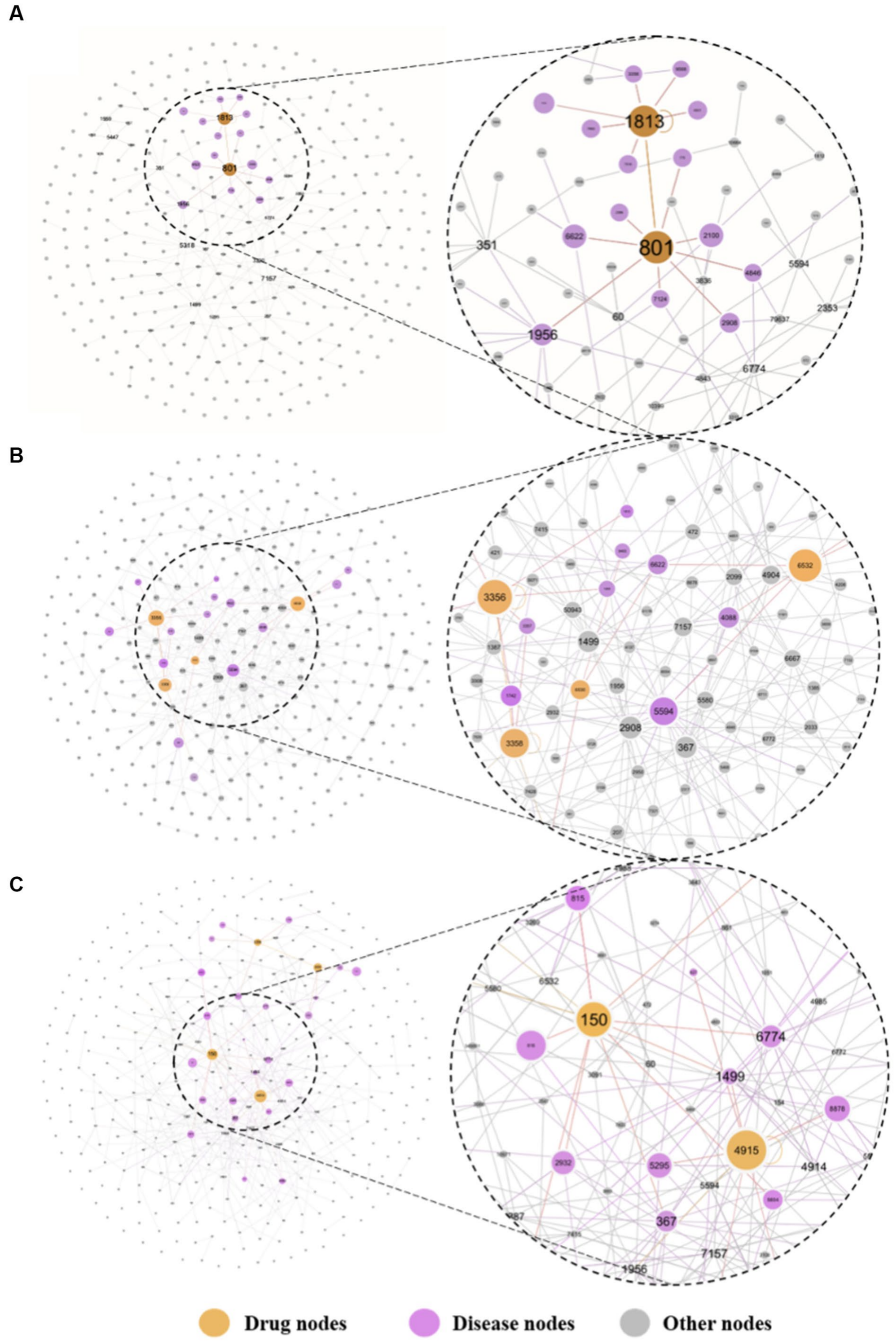
The key targeted relative maps are visualized between the top-3 drugs and depression. Where **(A)** presents the perphenazine-depression key target interaction map, **(B)** presents the clomipramine-depression key target interaction map, and **(C)** presents the amitriptyline-depression key target interaction map. The drugs at the top of the list were more closely associated with targets for depression. The undistorted images could be found in the supplementary materials.

Finally, we calculated the distance between 6,100 drugs and the depression disease module to rank them. The top 20 drug-disease proximity profiles are shown in Table 3. We found that most of the FDA-approved antidepressants were ranked highly. As shown in Figure 6, we mapped the network relationships between the first three drugs and the depressive disorders module. The drugs shown in Figures 6B,C are all FDA-approved antidepressants. We found that they have more acting relationships with the depressive disorder’s module in the network, which aligns with our expectations. Even more interesting is that these drugs usually also have anti-inflammatory effects, which suggests that depression and inflammation have potential similarities in terms of pathogenesis. This implies that anti-inflammatory strategies can alleviate depressive symptoms to some extent. In addition, drugs that treat other diseases and rank highly, we speculate that they may have a positive effect on the treatment of depression, although more clinical trials are needed to determine the results. We found that most of the FDA-approved drugs with antidepressant effects ranked high, and our analysis revealed that these drugs could usually treat not only depression but also diseases closely related to inflammation, such as cancer, chronic neuropathic pain, and diabetes. In other words, these drugs are likely to exert antidepressant effects by inhibiting inflammatory pathways or reducing levels of inflammatory factors. These drugs likely exert their antidepressant clinical effects by exerting anti-inflammatory effects. Thus, the mechanism by which antidepressants act is likely to be related to anti-inflammation, and there is a correlation between depression and inflammation. For example, among the top three drugs, Perphenazine is an antipsychotic phenothiazine derivative found in clinical studies to treat ear swelling mediated by 12-o-tetradecanoylphorbol-13-acetate (TPA) and oxazolone (OXA) and exert anti-inflammatory effects in addition to psychiatric disorders (28); Clomipramine is a tricyclic antidepressant that has been clinically found to also reduce LPS-induced neuroinflammation by partially modulating NLRP3 (29); Amitriptyline is also a tricyclic antidepressant, and many studies have found that Amitriptyline, in addition to its antidepressant and analgesic effects, can exert anti-inflammatory effects in humans and animal models of acute and chronic inflammation (30, 31). In addition to this many of the top-ranked drugs with antidepressant effects have been found to have some anti-inflammatory effects in clinical studies, and together these further assists in validating our prediction that there is a correlation between depression and inflammation. Second, by looking at the network action relationship map of disease-drug key targets, we found that most of the top-ranked drugs treated depression well and produced more associations with disease proteins in the network. This suggests that our method can measure the effect of drugs on the disease to some extent. It also suggests that drugs that are ranked high but are not currently used clinically as depression treatment are likely to be potentially effective for the treatment of depression, i.e., the indications for these drugs are likely to be similar to depression in terms of pathogenesis. More clinical trials are needed in the future to test our hypothesis.

**Table 3:** Top 20 drug-disease proximity.

Number	Drug	*Z*-value	Is it an antidepressant	Indication
1	Perphenazine	−9.633	No	**Perphenazine** is a phenothiazine used to treat schizophrenia as well as nausea and vomiting.
2	Clomipramine	−9.249	Yes	**Clomipramine** is a tricyclic antidepressant used in the treatment of obsessive–compulsive disorder and disorders with an obsessive–compulsive component, such as depression, schizophrenia, and Tourette’s disorder.
3	Amitriptyline	−9.236	Yes	**Amitriptyline** is a tricyclic antidepressant indicated in the treatment of depressive illness, either endogenous or psychotic, and to relieve depression associated anxiety.
4	Selegiline	−9.214	Yes	**Selegiline** is a monoamine oxidase inhibitor used to treat major depressive disorder and Parkinson’s.
5	Minaprine	−8.99	Yes	**Minaprine** is a psychotropic drug that has proved to be effective in the treatment of various depressive states.
6	Prasugrel	−8.744	No	**Prasugrel** is a P2Y12 platelet inhibitor used to reduce the risk of thrombotic cardiovascular events in unstable angina or non-ST-elevation myocardial infarction (NSTEMI), and in patients with STEMI when managed with either primary or delayed PCI.
7	Sertraline	−8.698	Yes	**Sertraline** is a selective serotonin reuptake inhibitor (SSRI) indicated to treat major depressive disorder, social anxiety disorder, and many other psychiatric conditions.
8	Sorafenib	−8.695	No	**Sorafenib** is a kinase inhibitor used to treat unresectable liver carcinoma, advanced renal carcinoma, and differentiated thyroid carcinoma.
9	Tranylcypromine	−8.631	Yes	**Tranylcypromine** is a monoamine oxidase inhibitor used to treat major depressive disorder.
10	Troglitazone	−8.59	No	**For** the treatment of Type II diabetes mellitus. It is used alone or in combination with a sulfonylurea, metformin, or insulin as an adjunct to diet and exercise.
11	Fluphenazine	−8.564	No	**Fluphenazine** is a phenothiazine used to treat patients requiring long-term neuroleptic therapy.
12	Duloxetine	−8.563	Yes	**Duloxetine** is a serotonin norepinephrine reuptake inhibitor used to treat generalized anxiety disorder, neuropathic pain, osteoarthritis, and stress incontinence.
13	Efavirenz	−8.521	No	**Efavirenz** is a non-nucleoside reverse transcriptase inhibitor used to treat HIV infection or prevent the spread of HIV.
14	Trimipramine	−8.488	Yes	**Trimipramine** is a tricyclic antidepressant used to treat depression.
15	Desipramine	−8.44	Yes	**Desipramine** is a tricyclic antidepressant used in the treatment of depression.
16	Meperidine	−8.396	No	**Meperidine** is an opioid agonist with analgesic and sedative properties used to manage severe pain.
17	Lopinavir	−8.395	No	**Lopinavir** is an HIV-1 protease inhibitor used in combination with ritonavir to treat human immunodeficiency virus (HIV) infection.
18	Esketamine	−8.323	Yes	**Esketamine** is a NMDA receptor antagonist used for treatment-resistant depression.
19	Pipotiazine	−8.243	No	**Pipotiazine** is an antipsychotic indicated for the management of chronic, non agitated schizophrenic patients.
20	Phenobarbital	−8.161	No	**Phenobarbital** is long-lasting barbiturate and anticonvulsant used in the treatment of all types of seizures, except for absent seizures.

The shortest path between the drug and the disease protein is calculated, and then the *Z*-value is calculated after eliminating the bias caused by the different number of targets of different drugs; the smaller the *Z*-value, the more effective the drug is.

Also importantly, we hope to reposition drugs by clarifying the inflammatory mechanisms of depression, i.e., “new use of old drugs.” Because drug development is often time-consuming, costly, and has a low success rate, new use of old drugs is a strategy to use old drugs or drugs in development for indications beyond the original approval and to expand their scope of application and use, which not only saves a lot of time and resources but also significantly accelerates the drug development process. Based on this scenario, we predict that the top-ranked, i.e., other drugs in the network with a similar distance to the depression disease module, may be useful for the treatment of depressed patients, and more reliable evidence will need to be provided by a large number of clinical trials.

## 4. Discussion

Diseases often occur not because of a defect in one gene but as a result of coordinated interactions between different genomes. In this paper, we examined the co-morbidity of depression based on the human protein interactome network, using a network analysis approach based on the depression disease module. We further investigated and predicted the correlation between depression and inflammation by calculating the distance between the depression disease module and other disease proteins, and by combining previous clinical studies to identify possible shared mechanisms between co-morbidities. A genetic study found that endothelial dysfunction and inflammation factors were definitively correlated with depressive symptoms in patients with heart disease (32). Similarly, the results of a clinical trial study assessing depression, metabolic syndrome, and inflammatory markers showed that increased appetite in depressed patients was positively correlated with CRP and HIF-α (33). The findings of this immunometabolism form of depression study suggest that we must be aware of disease co-morbidities in clinical treatment and consider immunometabolism and other pathways to intervene in depressive episodes. The important role of neuroinflammation in the pathogenesis of these co-morbidities suggests that there is likely a common feature between depression and these disorders, namely neuroimmune mechanisms. This suggests that we should pay attention to common disease co-morbidities in clinical practice to improve the treatment outcome.

Secondly, we screened critical targets based on the magnitude of target centrality, including the pro-inflammatory factors TNF-α and IL-6 and the anti-inflammatory factors IL-4 and IL-10. Numerous clinical studies have shown that depression is closely related to the concentration of inflammatory factors. Patients with depression usually have higher concentrations of pro-inflammatory factors such as TNF-α and IL-6 than healthy controls. As the disease progresses, their body usually has lower concentrations of the anti-inflammatory factor IL-10 than healthy controls (34). Studies have found that IL-6 concentrations are closely associated with depression and that the pro-inflammatory factor IL-6 may be involved in the brain inflammatory response through multiple pathways. Among others, an animal study showed that Toll-like receptor 4 genes and cytokine receptor genes are expressed in the choroid plexus (CP) and that stimulation of these receptors by cytokines induces the synthesis and eventual secretion of IL-6 into the blood-cerebrospinal fluid (CSF) (35). This, in turn, alters areas of the brain that regulate mood-related conditions and induces depressive symptoms. Through enrichment and pathway analysis of critical targets, we identified essential pathways related to hypoxia, inflammatory diseases, Etc. Several previous studies demonstrated the therapeutic effects of hypoxic preconditioning in a rat model of depression (36). In peripheral blood cells from depressed patients, studies likewise found that mRNA expression of HIF-1 and its target genes were mainly associated with depressive symptoms (37). In recent years, the hypoxic pathway that triggers neurodegenerative lesions has received attention. The hypoxia-inducible factor (HIF) plays a vital role in cellular biological processes and adaptation to cellular stress induced by hypoxic environments and is an essential transcriptional regulator. Therefore, we hypothesize that the HIF-1 signaling pathway is likely to play an important role in preventing depression. It also can help us identify and diagnose patients with atypical depression symptoms at the molecular level.

Finally, we ranked the drugs by finding a suitable algorithm to calculate the distance between drugs and disease modules after eliminating errors due to, for example, different numbers of drug targets. Looking at the top-ranked drugs and combining them with modern clinical studies, we found that most of the FDA-approved antidepressants also have anti-inflammatory effects, implying that the mechanism of action by which these drugs exert their antidepressant effects is likely to be the anti-inflammatory pathway at the same time. In an animal study, clomipramine was shown to attenuate lipopolysaccharide (LPS)-induced depression in a mouse model by partially modulating NLRP3 inflammatory vesicles (29). Among them, NLRP3 inflammatory vesicles are present in neurons and glial cells (38), which are involved in many critical innate immune processes, such as infection and inflammation. That is, NLRP3 inflammatory vesicles may be important in triggering depression. At the same time, we hypothesized that drugs with anti-inflammatory effects also have the potential to exert antidepressant efficacy, and the results of a systematic evaluation of the efficacy and safety of anti-inflammatory drugs in patients with major depression showed that anti-inflammatory drugs not only help to treat depressed patients and exert antidepressant effects but also are quite safe (39).

In conclusion, it is reasonable to conclude that there is a strong correlation between depression and inflammation, whether from the perspective of co-morbidity, signaling pathway analysis, or antidepressant drug action analysis, which provides a basis for the inclusion of anti-inflammatory strategies in the treatment of depression in the future and provides a feasible approach to explore the inflammatory mechanisms of depression. It also provides some reference ideas for new applications of old drugs to complex diseases. This has some applicability in both clinical treatment strategies and drug development.

There are several limitations to this study. Our study is based on network predictions, and more clinical practice is needed to explore the relationship between neuroinflammation and the onset of depression and its specific mechanisms of action. In addition, we only focused on the use of the drug without refining the therapeutic effects and adverse effects. Therefore, further studies are necessary to address these issues and provide a more comprehensive evaluation of their efficacy and safety. Despite these limitations, our study provides preliminary insights into the potential role of anti-inflammatory drugs as a treatment for depression and merits further investigation.

## Data availability statement

The original contributions presented in the study are included in the article/Supplementary materials, further inquiries can be directed to the corresponding author.

## Author contributions

YZ: conceptualization, funding acquisition, and supervision and project administration. YZ, HP, and JL: methodology. HP: formal analysis. XH: resources. YW: suggestion. XH, HP, YZ, and YL: writing—original draft. XH, HP, and JL: visualization. All authors contributed to the article and approved the submitted version.

## Funding

This study was supported by Scientific and Technological Innovation Project of China Academy of Chinese Medical Sciences (No. C12021A05042, No. CI2021A05401), the National Natural Science Foundation of China (No. 81674101), National Key Technology Support Program (No. 2012BAI25B02), Self-selected subject of China Academy of Chinese Medical Sciences (No. Z0217).

## Conflict of interest

The authors declare that the research was conducted in the absence of any commercial or financial relationships that could be construed as a potential conflict of interest.

## Publisher’s note

All claims expressed in this article are solely those of the authors and do not necessarily represent those of their affiliated organizations, or those of the publisher, the editors and the reviewers. Any product that may be evaluated in this article, or claim that may be made by its manufacturer, is not guaranteed or endorsed by the publisher.

## Footnotes

^1^ https://www.ncbi.nlm.nih.gov/mesh/

^2^ https://www.genecards.org/

^3^ http://ctdbase.org/

^4^ https://www.uniprot.org/

^5^ https://string-db.org/
