# Supplementary figures and images for: A network medicine-based approach to explore the relationship between depression and inflammation
Source: Front Psychiatry. 2023 Jul 10;14:1184188. doi: 10.3389/fpsyt.2023.1184188 (PMC10364440; doi:10.3389/fpsyt.2023.1184188)

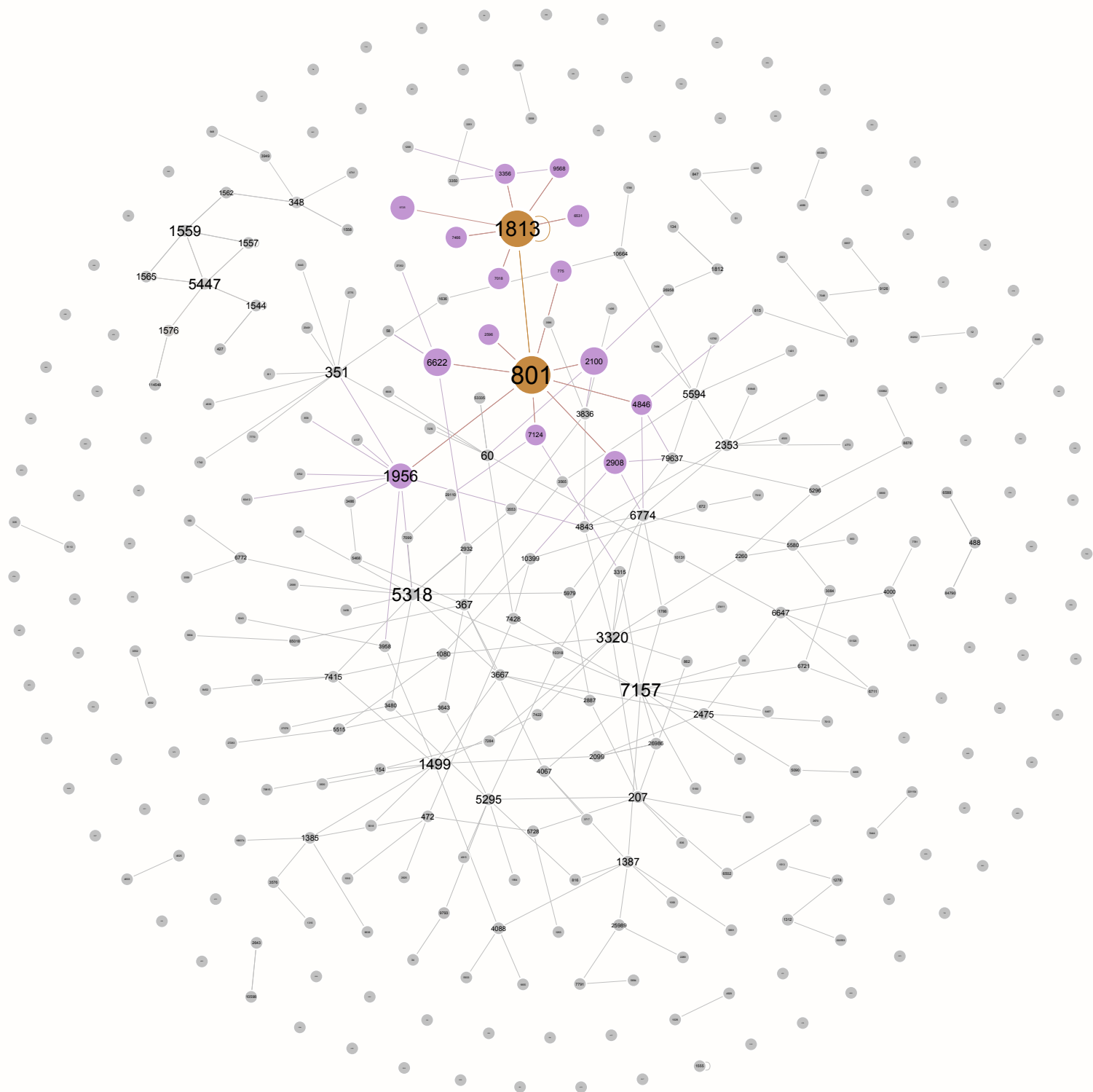

Supplement: Supplementary file 1 [file Data_Sheet_1.PDF]

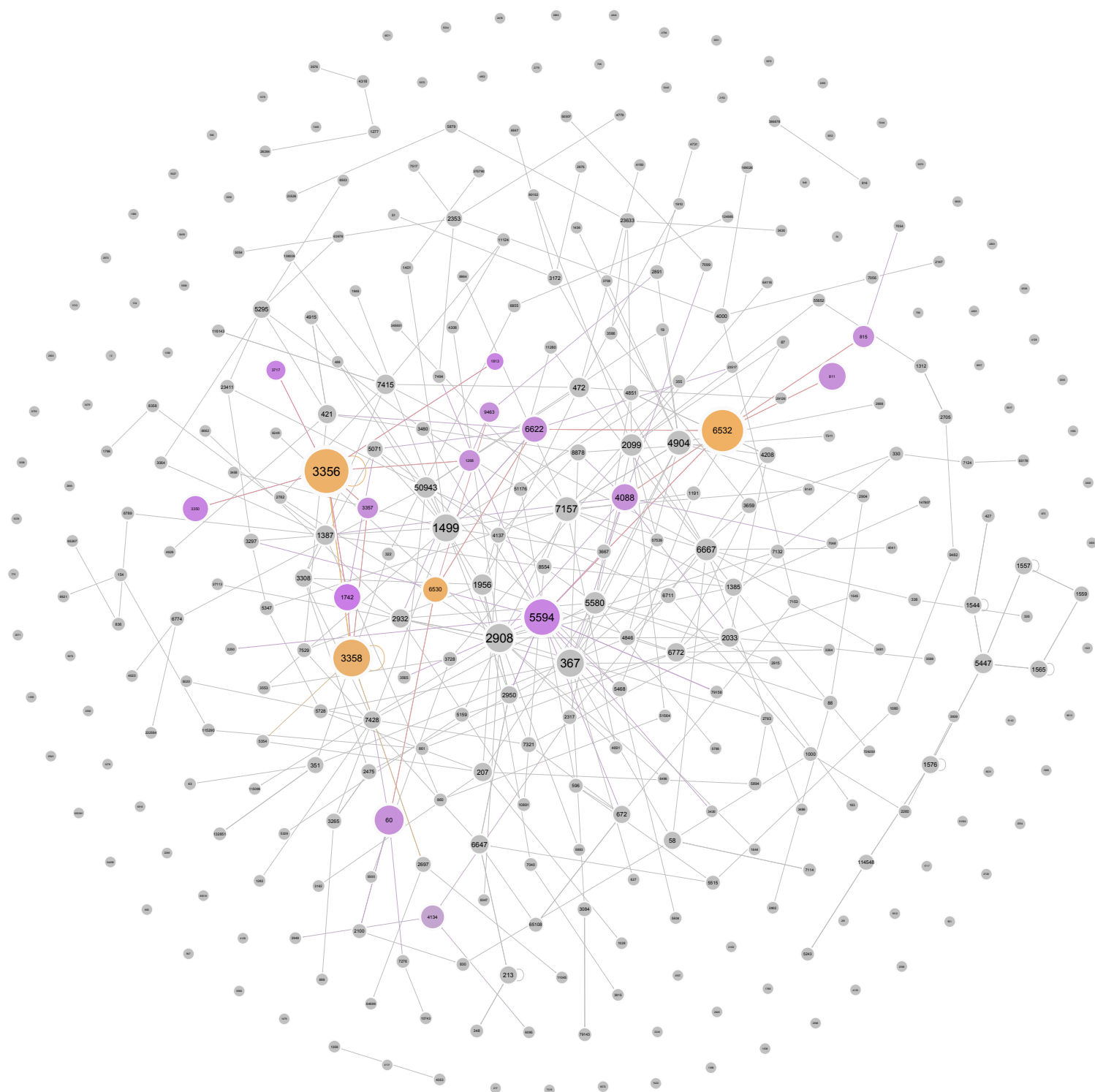

Supplement: Supplementary file 2 [file Data_Sheet_2.PDF]

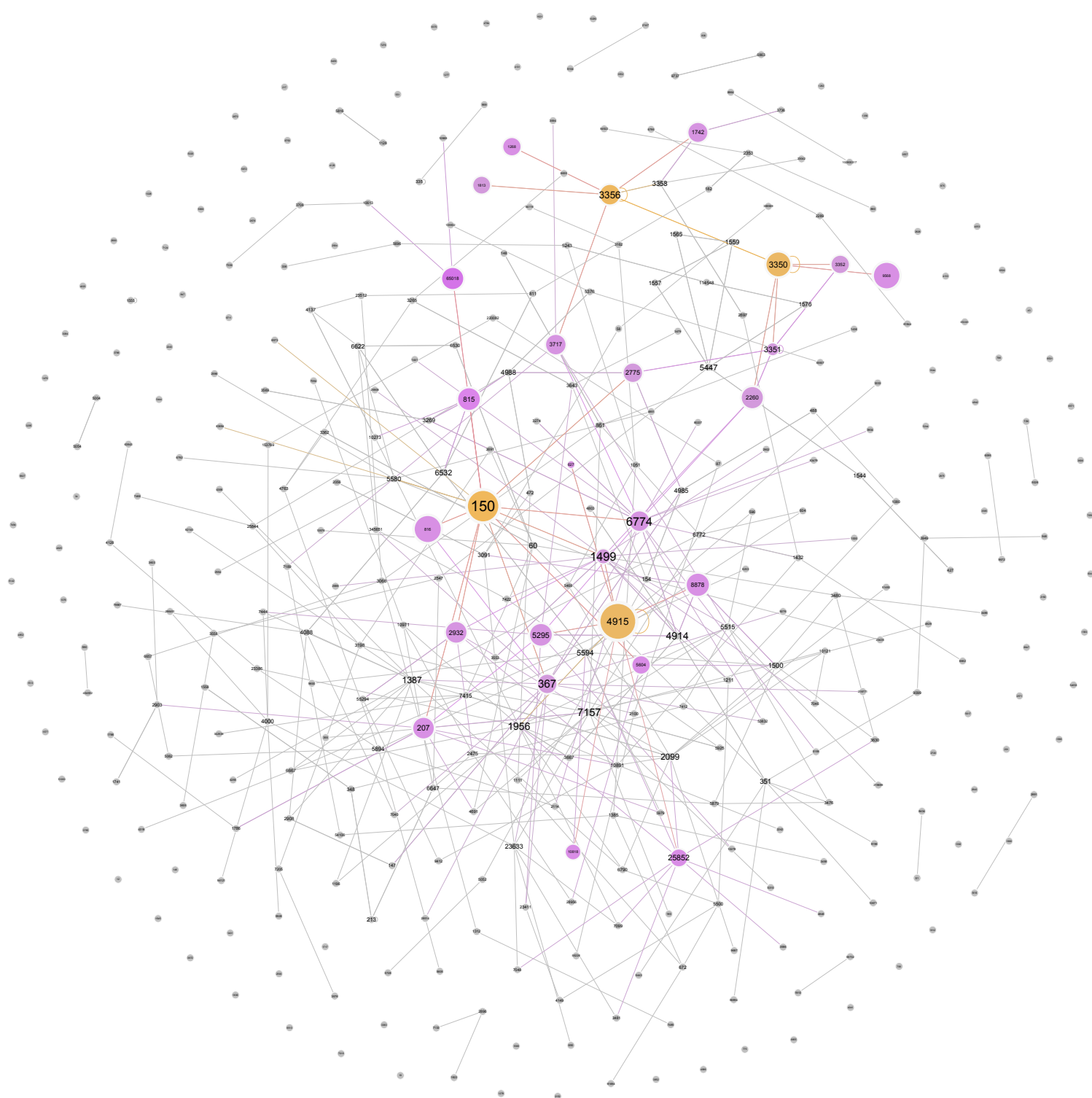

Supplement: Supplementary file 3 [file Data_Sheet_3.PDF]

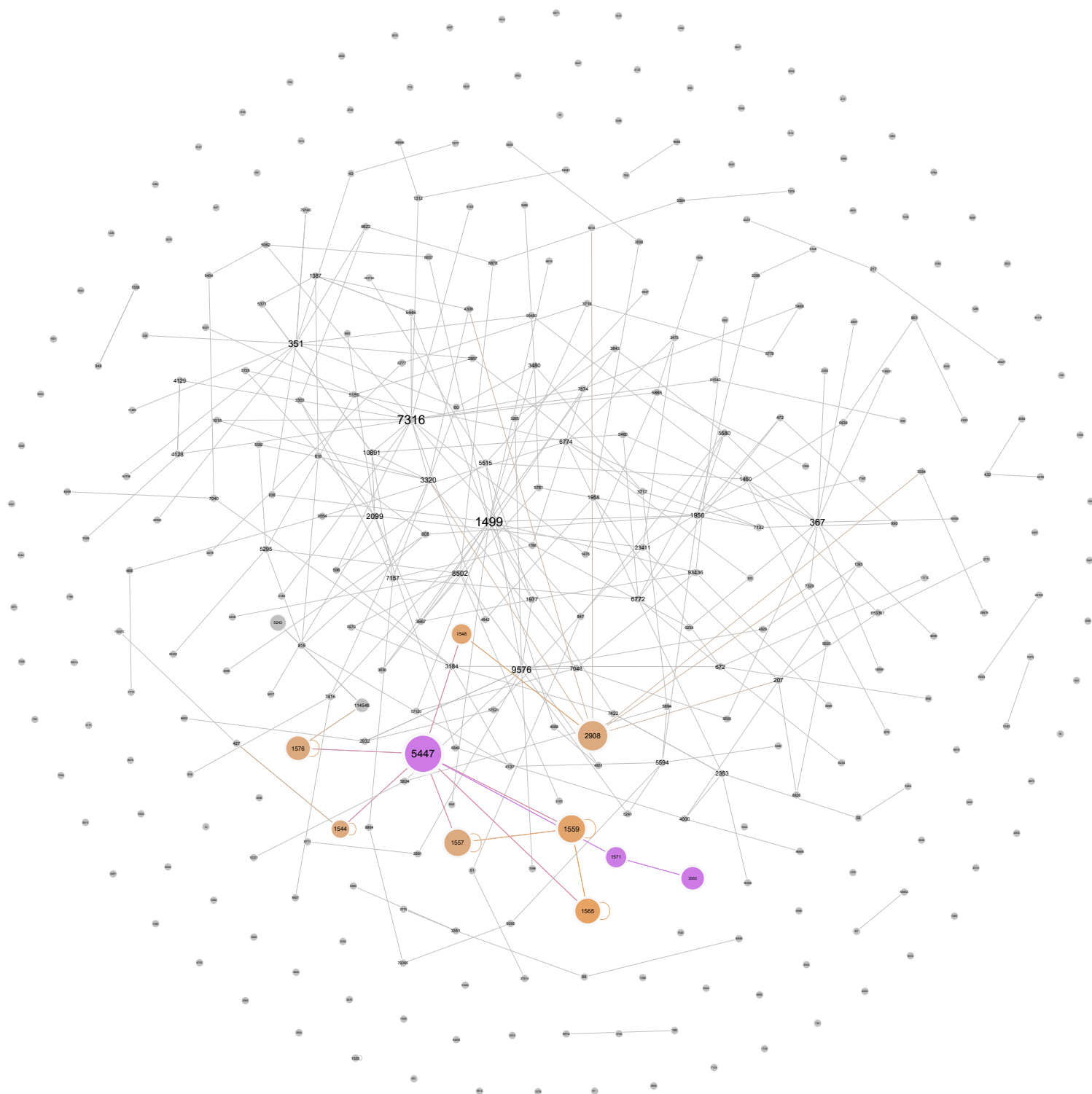

Supplement: Supplementary file 4 [file Data_Sheet_4.PDF]

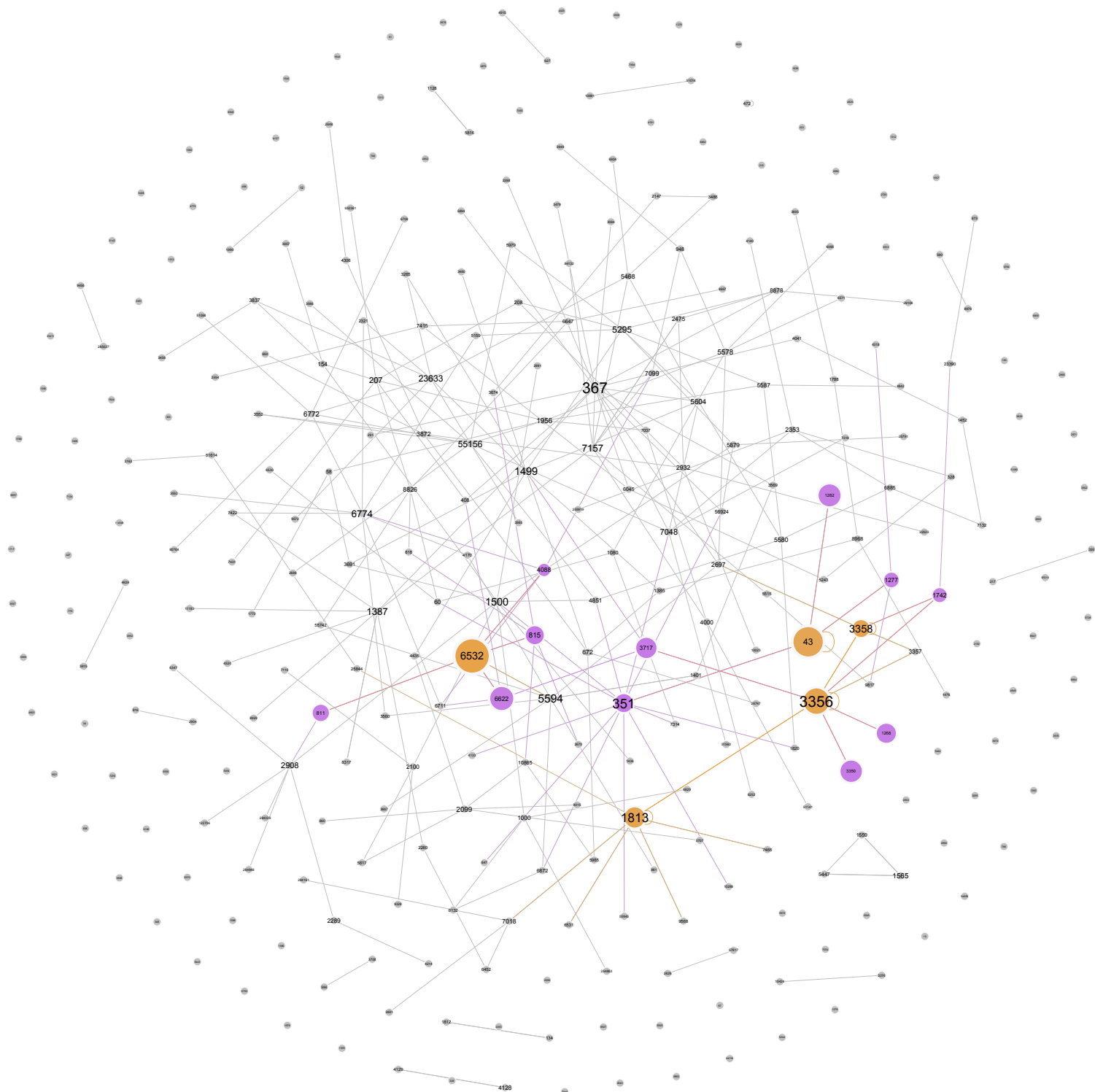

Supplement: Supplementary file 5 [file Data_Sheet_5.PDF]
